# Supplementary figures and images for: Genomic profiling of active vitamin D colonic responses in African- and European-Americans identifies an ancestry-related regulatory variant of POLB
Source: PLoS Genet. 2026 Jan 8;22(1):e1011983. doi: 10.1371/journal.pgen.1011983 (PMC12810902; doi:10.1371/journal.pgen.1011983)

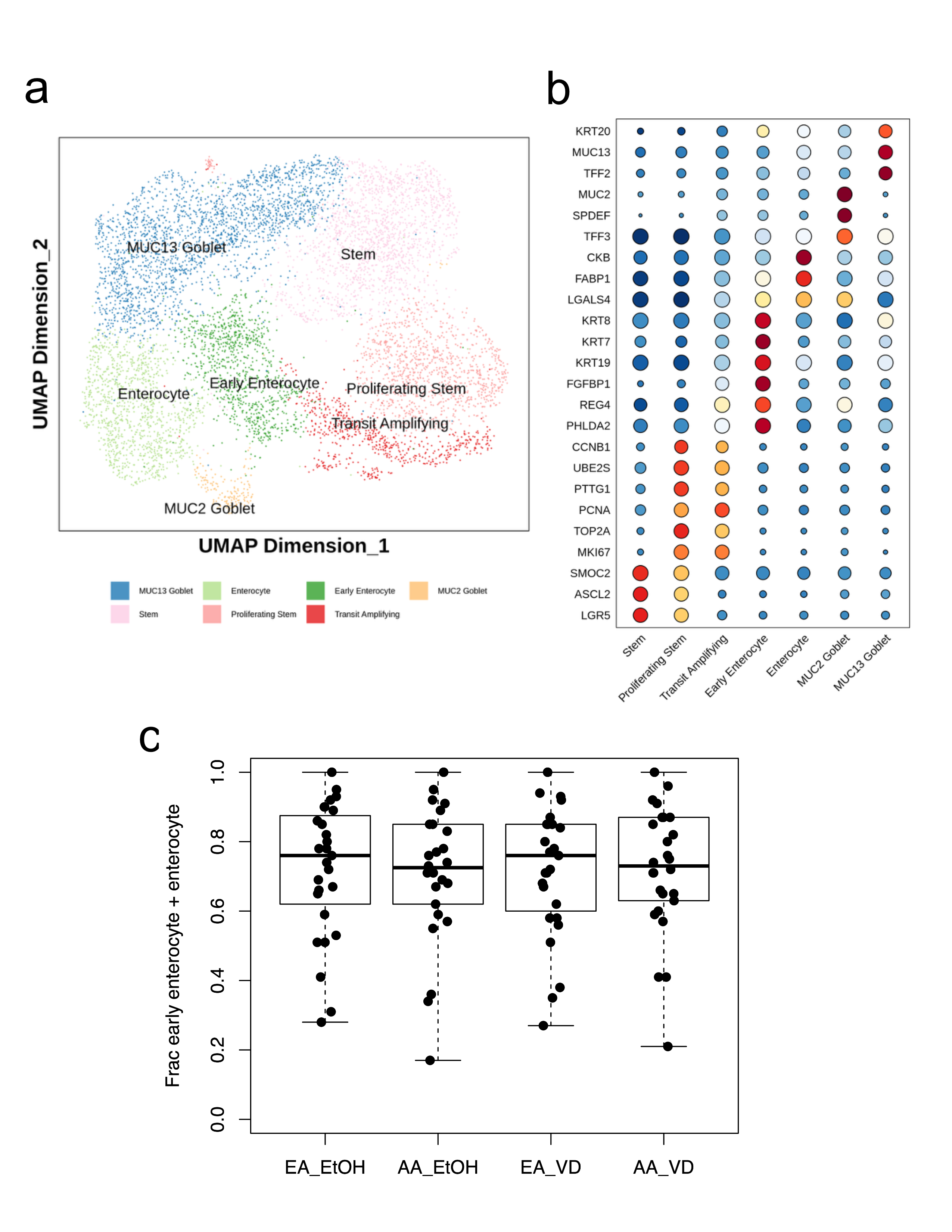

Supplement: S1 Fig — As described in the Methods, organoids from a single individual were cultured in growth and differentiation media for 24, 48 and 72 hours and single cell RNA-sequencing was performed. Data was utilized to assess cell composition measured as the fraction of early enterocytes and enterocytes for downstream analyses in this study. a) Uniform Manifold Approximation and Projection (UMAP) of single cell sequencing. The UMAP plot shows 7 clusters of cell types. b) Cell type markers. Clusters were annotated based on expression of cell type marker genes previously reported in the literature (see references for genes in Methods). c) Percentage of early enterocytes and enterocytes by treatment and population. The percentage of early enterocytes and enterocytes was used to control for cell composition in downstream analyses. There were no differences by treatment or population. (TIFF) [file pgen.1011983.s001.tiff]

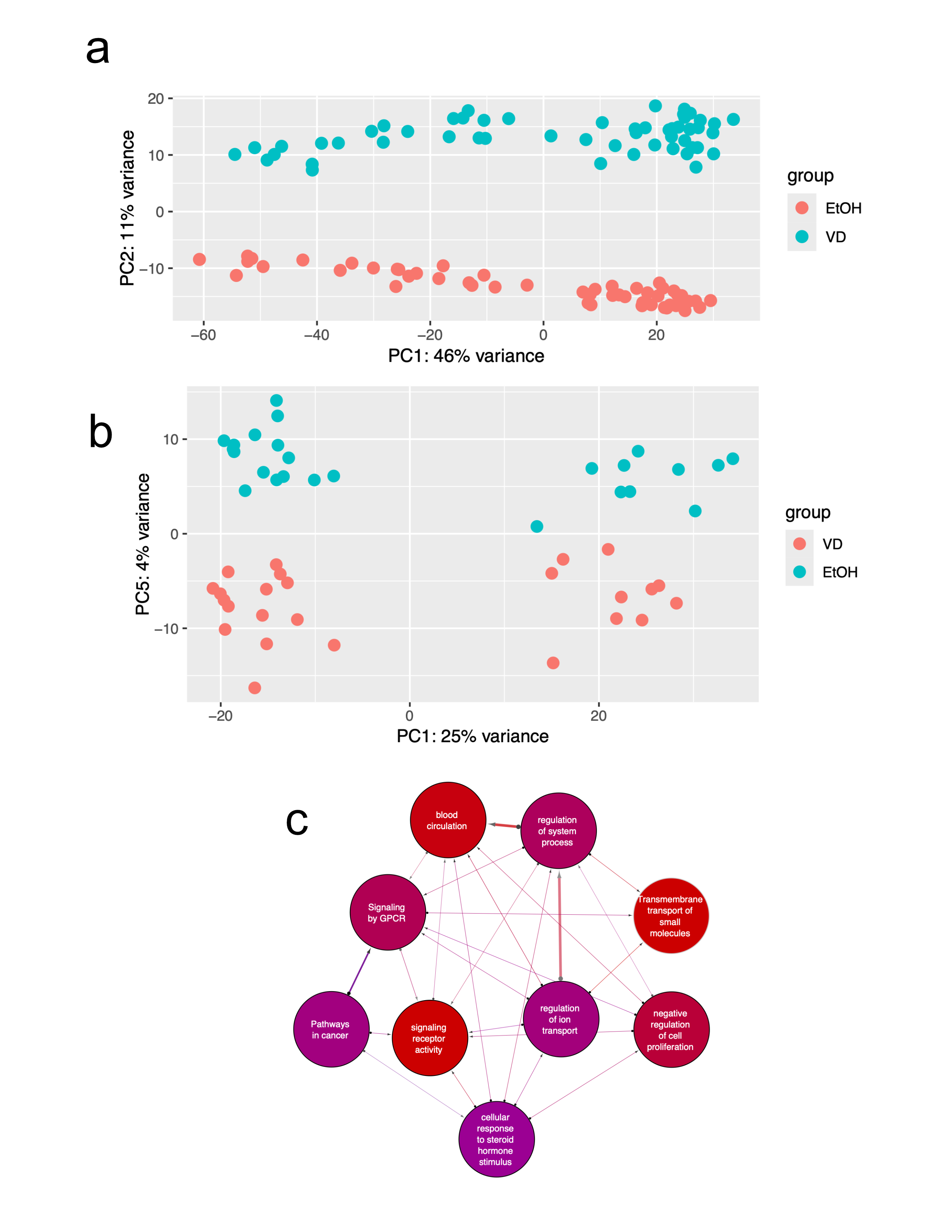

Supplement: S2 Fig — VD transcriptional responses measured by RNA-seq were separated by PC2, which accounted for 11% of the variance. b) PC plot of chromatin accessibility responses. VD chromatin accessibility responses measured by ATAC-seq were separated by PC5, which accounted for 4% of the variance. Separation along PC1 represents variation due sex-linked chromatin accessibility. c) SetRank network plot. To visualize interactions of top enriched pathways of DE genes, we used the network output of SetRank plotted using the program Cytoscape (v3.10.2). We show the interactions between the only disease-associated enriched pathway called “pathways in cancer” (KEGG hsa05200) with the top enriched pathways. The node fill color reflects the SetRank corrected p-values with blue to red indicating decreasing p-values. The edge arrows represent interaction from least significant gene set to more significant gene set. (TIFF) [file pgen.1011983.s002.tiff]

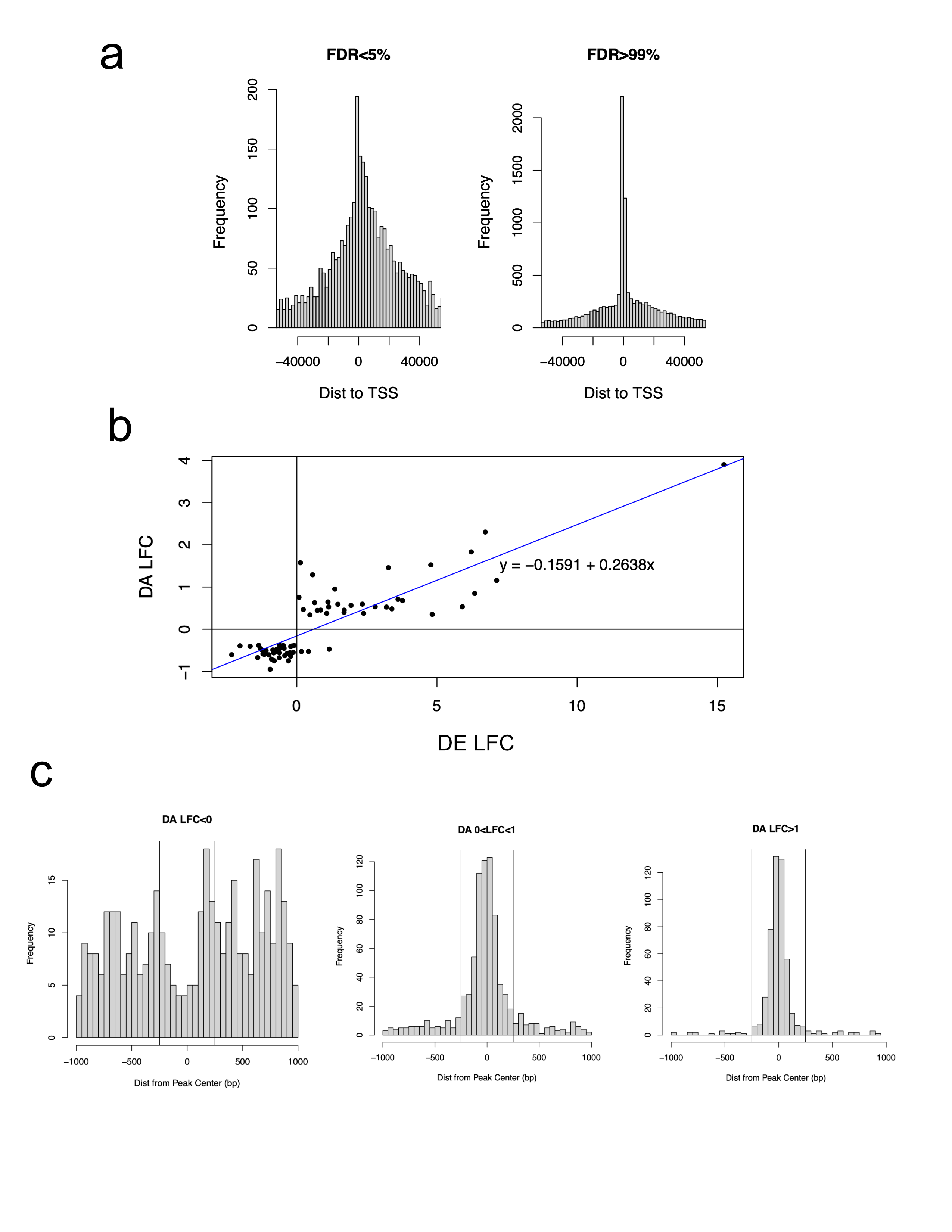

Supplement: S3 Fig — For DA peaks (FDR < 5%), a broader distribution of distances to the transcription start site (TSS) of the nearest gene was observed relative to peaks that were not DA (FDR > 99%). This observation is in line with the finding that DA peaks were significantly depleted in promoter regions (3.6% vs. 12.4%, respectively; hypergeometric test p = 1.54x10^-89^) compared to intronic or intergenic regions. b) Correlation of DE genes and DA peaks in promoter regions. A total of 85 tested protein-coding genes were found to have DA peaks falling in their promoters. Of these, 73/85 (86%) were DE, a 1.3-fold enrichment over genes without DA peaks in their promoters (hypergeometric test p = 8.18x10^-5^). The corresponding DE and DA effect sizes for these 73 genes were strongly correlated (r = 0.85; p < 2.2x10^-16^). c) Vitamin D response element (VDRE) peak enrichment. The VDRE motif was found within 250 bp of a DA peak center for 75% of DA peaks with large effects (i.e., LFC > 1), 46% with moderate effects (i.e.,0 < LFC < 1), and only 3.8% of DA peaks with reduced effects (i.e., LFC < 0). These patterns were similar to VDR enrichment patterns. (TIFF) [file pgen.1011983.s003.tiff]

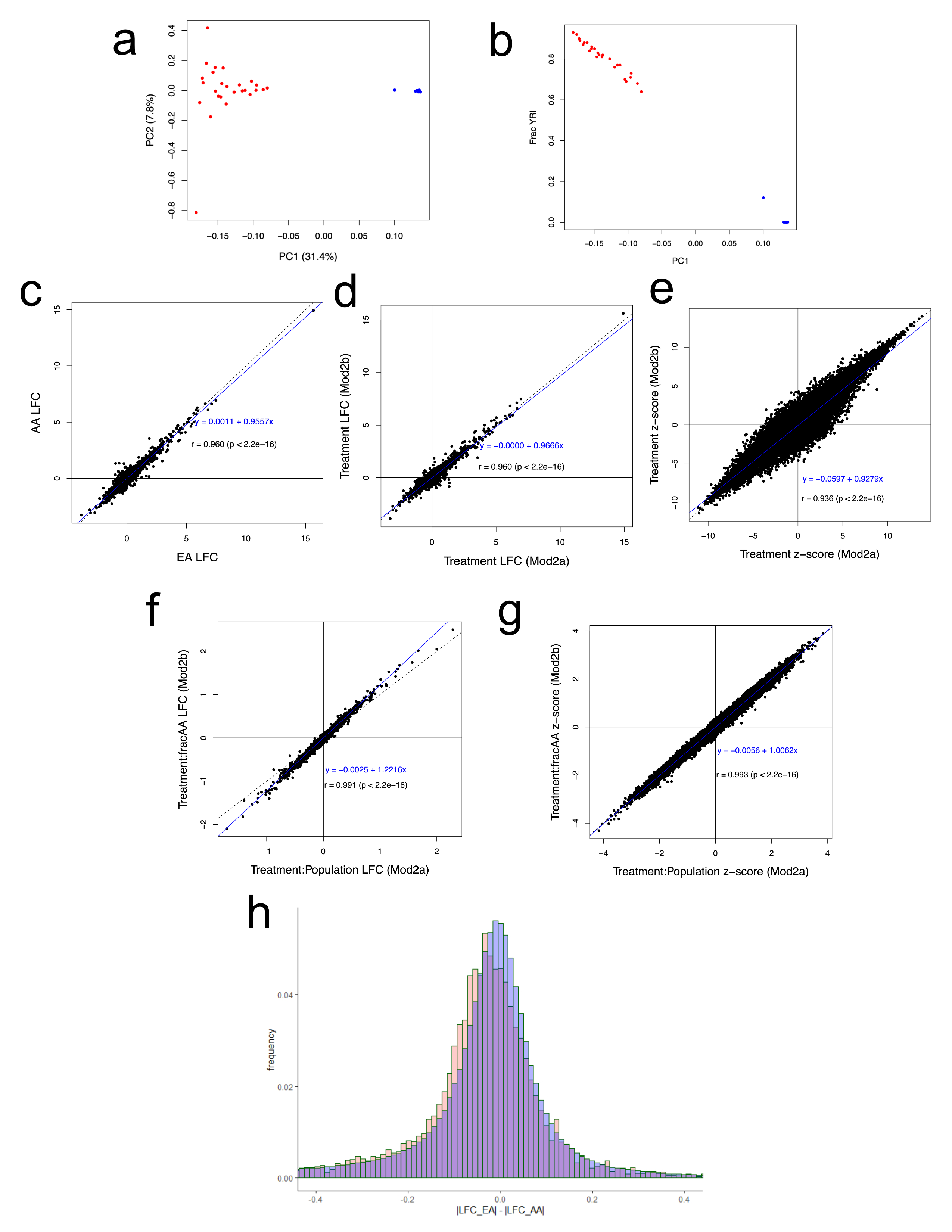

Supplement: S4 Fig — a) Principal component PC1 vs PC2 of genotype SNP data with self-identified White in blue dots and self-identified African-American in red dots, and b) PC1 vs fraction of African (i.e., YRI) ancestry with self-identified White in blue dots and self-identified African-American in red dots. Genetic ancestry proportions were estimated with the program ADMIXTURE (v1.3.0) using approximately 255,000 imputed SNPs. c) Correlation of transcriptional response effect sizes in AA and EA lines (Model 2a). There was significant correlation between effect sizes (i.e., LFC) for DE genes with treatment between AA and EA (r = 0.960; p < 2.2 x 10^-16^). This result was similar to results for z-scores shown in Fig 1e. d-g) Correlation of treatment effects between models that include fraction African ancestry (Model 2b) and self-identified race (Model 2a). We assessed treatment responses using both fraction African ancestry and self-identified race. Comparison of both effect sizes and z-scores for the treatment and interactions terms of the models showed very high correlations and similar power. For the interaction term, there was near perfect correlation between results from the two models (r > 0.99; p < 2.2 x 10^-16^). h) Genome-wide transcriptional responses by ancestry. To determine whether there were differences in overall absolute response to 1,25D treatment, we compared the between population transcriptional effect size magnitude difference (|LFCAA| - |LFCEA|) to the expected null distribution generated from permuted samples. Using this approach, neither population showed a stronger overall genome-wide response to treatment (p = 0.28). (TIFF) [file pgen.1011983.s004.tiff]

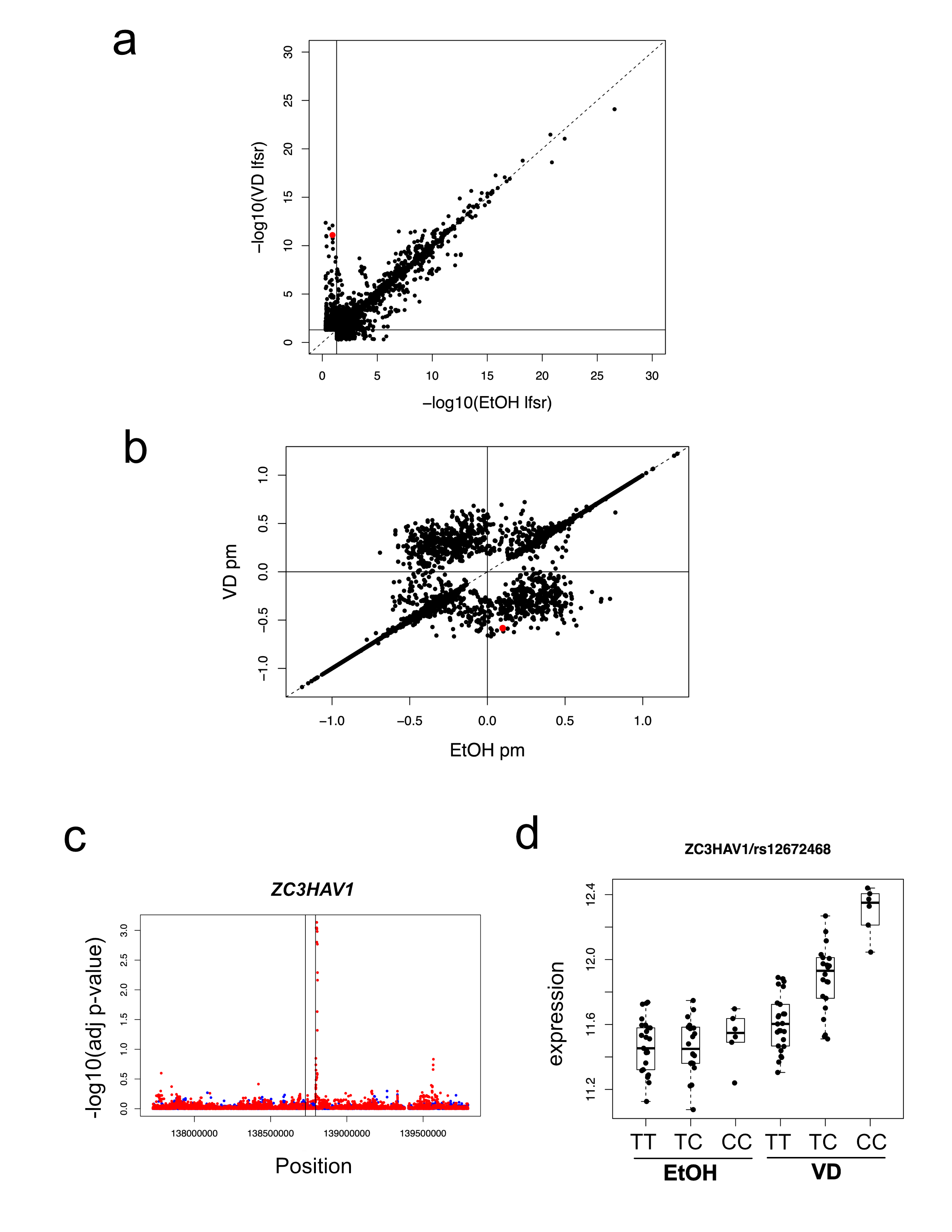

Supplement: S5 Fig — a) mashr local false sign rate (lfsr) for variants in 1,25D (y-axis) versus control (x-axis) showing rs2272733-POLB (red dot). Dashed line represents y = x. b) mashr effect size posterior mean (pm) for variants in VD (y-axis) versus control (x-axis) showing rs2272733-POLB eQTL (red dot). Dashed line represents line y = x. c-d) ZC3HAV1 eQTLs. c) MatrixEQTL adjusted p-values were plotted as a function of physical position for variants within a 2 Mb window centered on ZC3HAV1 for VD (red dots) or control (blue dots) treatment conditions. Vertical lines represent position of gene. d) ZC3HAV1 shows association with rs12672468 genotype only in 1,25D treatment condition. (TIFF) [file pgen.1011983.s005.tiff]

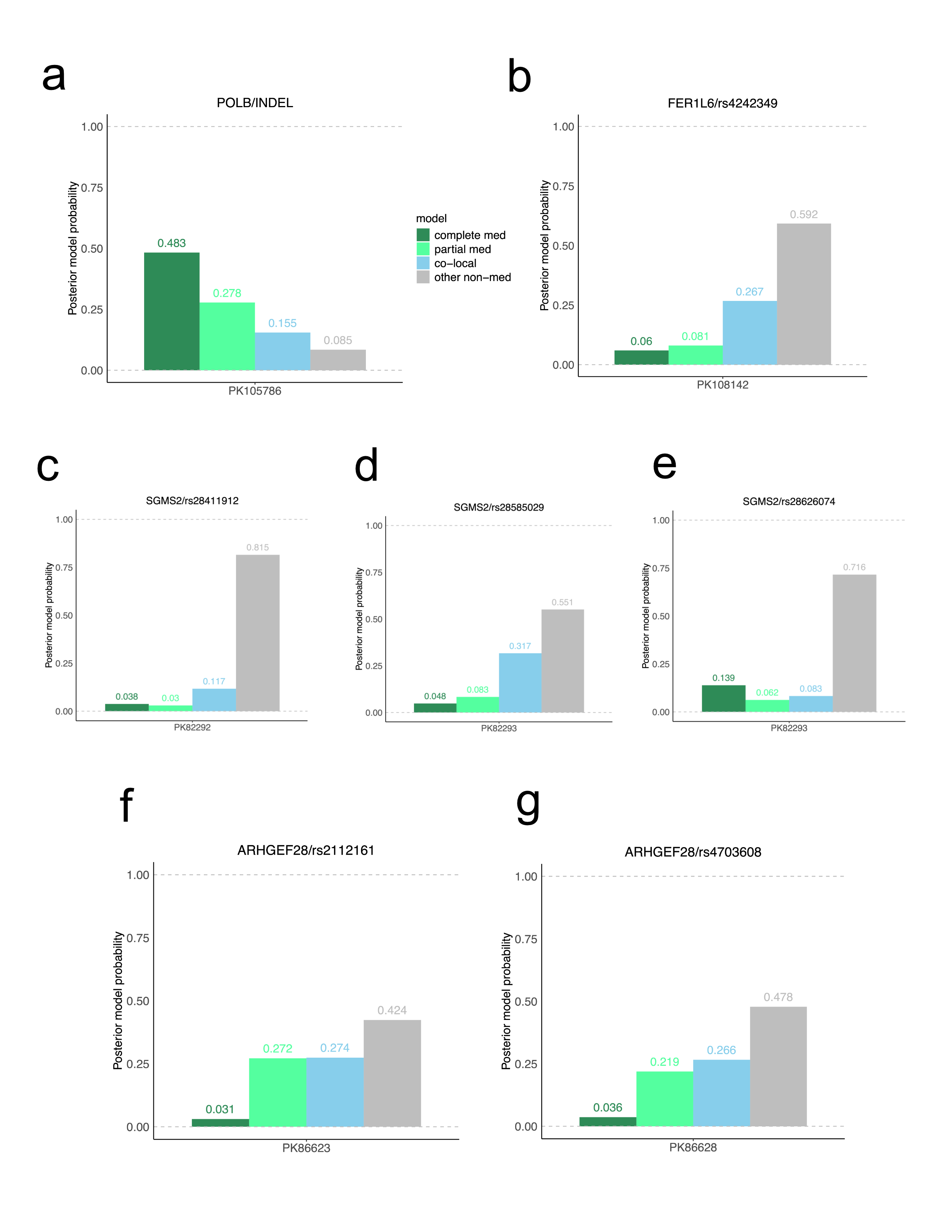

Supplement: S6 Fig — bmediatR assessed 4 models of reQTL mediation by a DA peak and showed a high posterior probability for complete or partial mediation only for the indel-POLB reQTL. a) 8:42190387:A: < CN0 > :42194352-PK105786-POLB. b) rs4242349-PK108142-FER1L6. c) rs28411912-PK82292-SGMS2. d) rs28585029-PK82293-SGMS2. e) rs28626074-PK82293-SGMS2. f) rs2112161-PK86623-ARHGEF28. g) rs4703608-PK86628-ARHGEF28 (TIFF) [file pgen.1011983.s006.tiff]

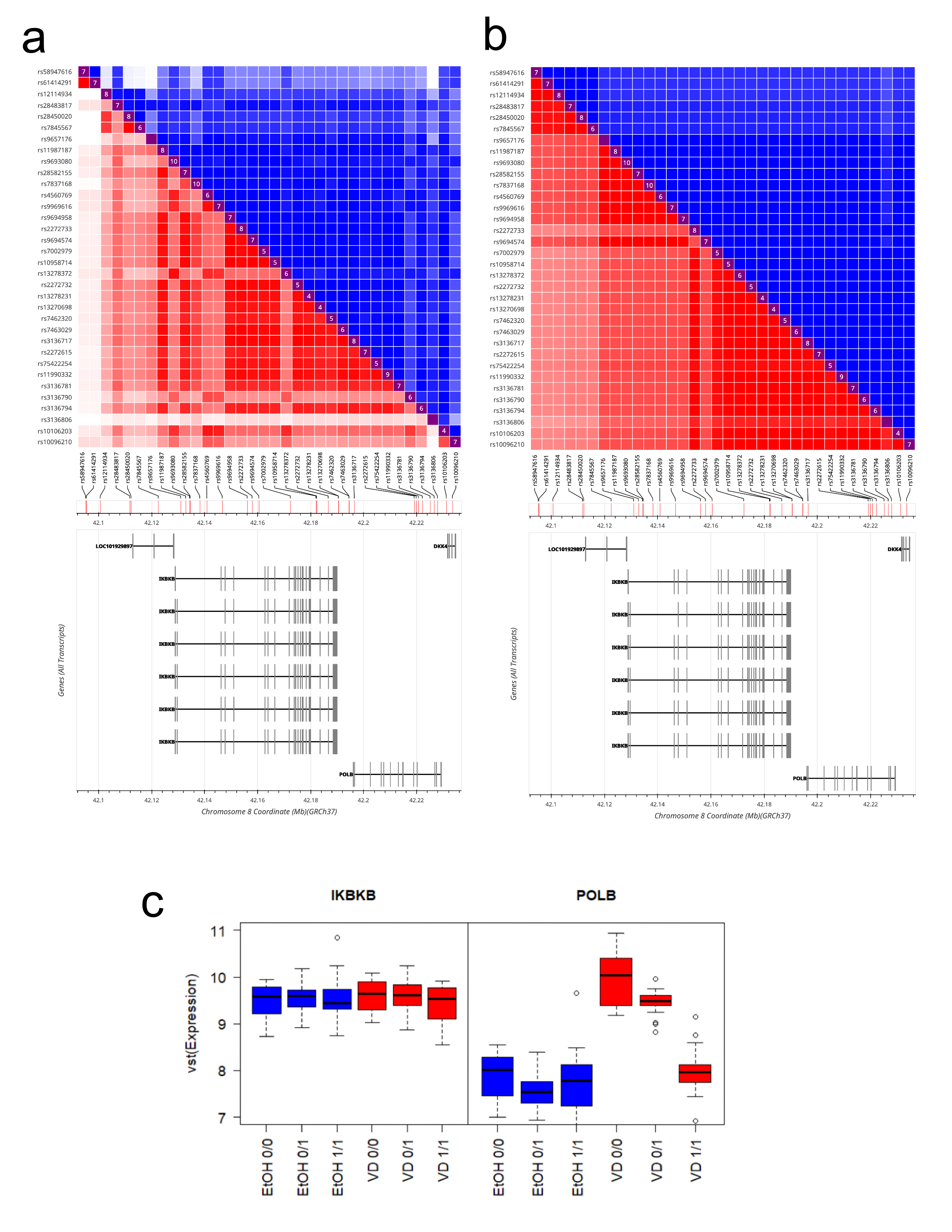

Supplement: S7 Fig — Of the 33 SNPs that were daQTLs and POLB reQTLs, 10 SNPs (rs2272733, rs7002979, rs10958714, rs13278231, rs13270698, rs7462320, rs7463029, rs3136717, rs75422254, rs11990332) were found to define a core haplotype where pairwise SNP r2 > 0.90 in both 4a) YRI and 4b) CEU 1KGP populations. c) Core haplotype spans POLB and IKBKB. The core haplotype spans the genes POLB and IKBKB. The tagging SNP rs2272733 showed no association with IKBKB expression nor did IKBKB show a differential response to 1,25 vitamin D treatment, while POLB showed an association with genotype only in response to 1,25 vitamin D treatment. Expression shown as the DESeq2 variance stabilized transformation (vst) of normalized read counts. (TIFF) [file pgen.1011983.s007.tiff]

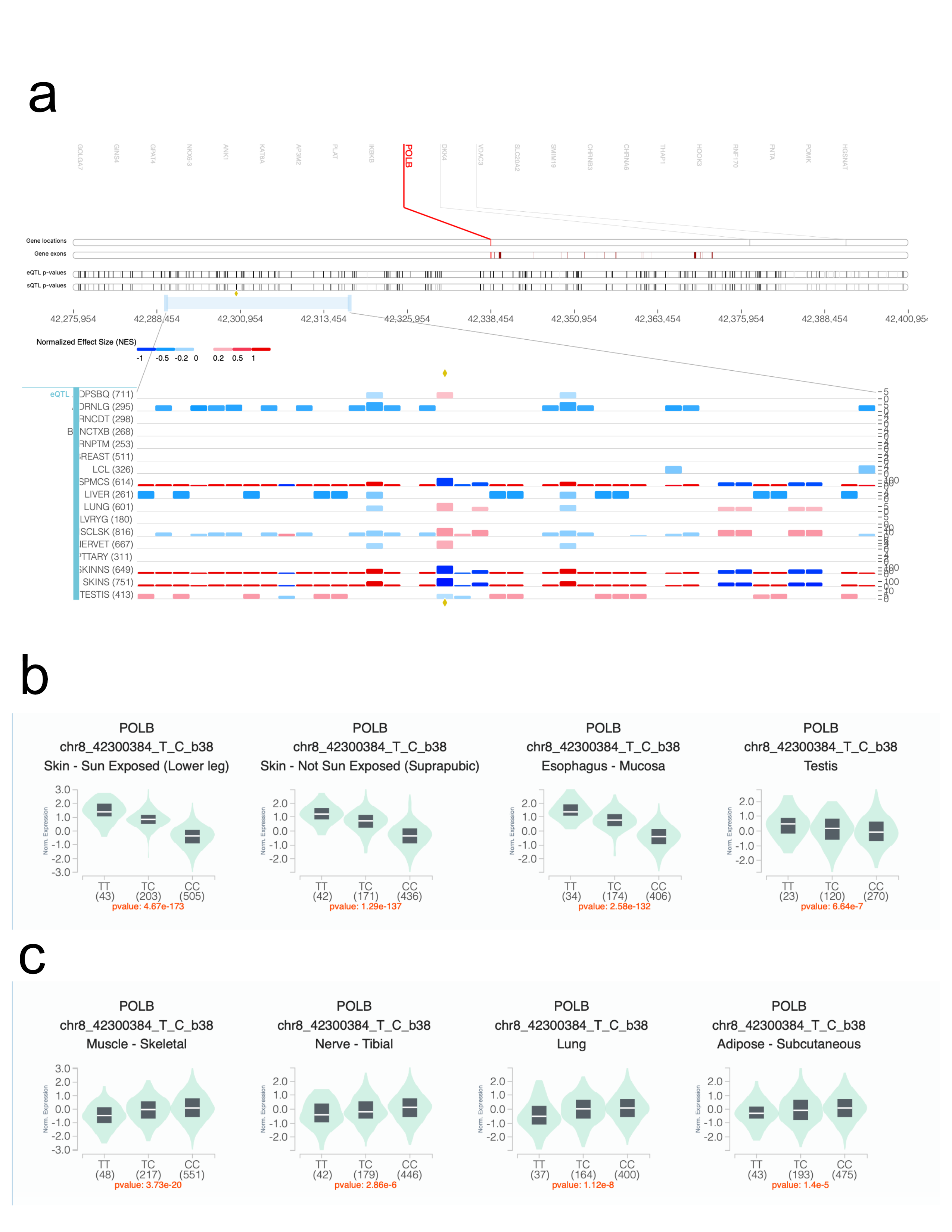

Supplement: S8 Fig — The effects of rs2272733 on POLB expression differ by tissue type. Shown here are GTEx tracks for different tissues showing effect sizes by color. rs2272733 is shown as the vertical line. b) rs2272733 cis-eQTL effects on POLB in the same direction as 1,25 vitamin D colonic responses. Tissues that showed similar direction of POLB cis-eQTL effects for rs2272733 included skin (sun exposed and non-sun exposed), esophagus and, to a lesser extent, testis. c) rs2272733 cis-eQTL effects on POLB in the opposite direction as 1,25 vitamin D colonic responses. Tissues that showed opposite direction of POLB cis-eQTL effects for rs2272733 included skeletal muscle, nerve, lung and subcutaneous adipose tissue. (TIFF) [file pgen.1011983.s008.tiff]
