# Supplementary material for: Genomic profiling of active vitamin D colonic responses in African- and European-Americans identifies an ancestry-related regulatory variant of POLB
Source: PLoS Genet. 2026 Jan 8;22(1):e1011983. doi: 10.1371/journal.pgen.1011983 (PMC12810902; doi:10.1371/journal.pgen.1011983)
Supplement: S1 Methods — (DOCX) [file pgen.1011983.s018.docx]

**Supplementary Methods**

***Single cell sequencing****.* In a previous experiment, colonic organoids from a single individual were treated in growth and differentiation media for 24, 48 and 72 hours. After culturing, the MULTI-Seq barcoding and library preparation protocol [1] was followed that utilizes the Single Cell 3’ reagent kit (10x Genomics). Briefly, single cell suspensions were prepared and barcoded by sequentially adding anchor:barcode solution, incubating on ice, followed by the addition of co-anchor solution and further incubation. Cell samples were transferred to individual wells and washed by centrifugation with 1% BSA in PBS. Barcoded cells were pooled, filtered through a cell strainer, and counted. Downstream processing followed the 10X Genomics single-cell RNA-sequencing workflow. cDNA amplification was performed using a master mix containing MULTI-seq primer, followed by a 0.6X SPRI clean-up, retaining the supernatant as the barcode fraction. Barcode DNA was further purified via magnetic bead-based extraction, washed, eluted, and quantified using Qubit fluorometric analysis. A PCR master mix was prepared for barcode library amplification and subjected to cycling conditions: initial denaturation, 8-12 cycles of amplification, and a final extension. The resulting products were purified with SPRI beads, washed with ethanol, air-dried, and eluted. Barcode library quality and concentration were assessed with Agilent Bioanalyzer High Sensitivity DNA analysis. Libraries were sequenced 100 base pair paired-end (R1: 30 cycles, I1: 10 cycles, R2: 85 cycles) on an Illumina NovaSeq 6000 in the Functional Genomics core at University of Chicago to an average depth of 75,297 mean reads per cell.

***Single cell data analysis.*** The cell ranger count pipeline of the 10X Genomics Cell Ranger (v8.1.0) software package [2] was used to align reads to the transcriptome (refdata-gex-GRCh38-2020-A) and to generate matrices of UMI counts for each feature. The program estimated 12,740 cells with a mean of 75,285 reads per cell and a median of 4,834 genes per cell. The Cell Ranger count matrix was then imported into the Seurat [3](v4.1.0) R package where the data were filtered using the parameters of the standard pre-processing workflow (cells were retained if 1000 < gene count < 5000 genes and if MT count < 10%) and then normalized. Seurat was then used to perform cell clustering using the UMAP embeddings. Cluster (**Supplementary Figure 1**) were annotated based on expression of cell type markers previously reported in the literature: stem (*LGR5[4], ASCL2[5], SMOC2[6, 7])*, proliferating stem (*TOP2A[8]*) transit amplifying (*PCNA[9]*), early enterocyte (*PHLDZ2[10], REG4[11], FGFBP1[12], KRT7,8, 19[13])*, enterocyte (*FABP1[14]*), goblet (*MUC2[15], SPDEF[16]*), and a cluster marked by *MUC13[17, 18]*, *KRT20[19]* and *TFF2[20]*.

**Supplementary References**

1. McGinnis CS, Patterson DM, Winkler J, Conrad DN, Hein MY, Srivastava V, et al. MULTI-seq: sample multiplexing for single-cell RNA sequencing using lipid-tagged indices. Nat Methods. 2019;16(7):619–26. Epub 20190617. doi: 10.1038/s41592-019-0433-8. PubMed PMID: 31209384; PubMed Central PMCID: PMCPMC6837808.

2. Zheng GX, Terry JM, Belgrader P, Ryvkin P, Bent ZW, Wilson R, et al. Massively parallel digital transcriptional profiling of single cells. Nat Commun. 2017;8:14049. Epub 20170116. doi: 10.1038/ncomms14049. PubMed PMID: 28091601; PubMed Central PMCID: PMCPMC5241818.

3. Hao Y, Hao S, Andersen-Nissen E, Mauck WM, 3rd, Zheng S, Butler A, et al. Integrated analysis of multimodal single-cell data. Cell. 2021;184(13):3573–87 e29. Epub 20210531. doi: 10.1016/j.cell.2021.04.048. PubMed PMID: 34062119; PubMed Central PMCID: PMCPMC8238499.

4. Carmon KS, Lin Q, Gong X, Thomas A, Liu Q. LGR5 interacts and cointernalizes with Wnt receptors to modulate Wnt/beta-catenin signaling. Mol Cell Biol. 2012;32(11):2054–64. Epub 20120402. doi: 10.1128/MCB.00272-12. PubMed PMID: 22473993; PubMed Central PMCID: PMCPMC3372227.

5. van der Flier LG, van Gijn ME, Hatzis P, Kujala P, Haegebarth A, Stange DE, et al. Transcription factor achaete scute-like 2 controls intestinal stem cell fate. Cell. 2009;136(5):903–12. doi: 10.1016/j.cell.2009.01.031. PubMed PMID: 19269367.

6. Merlos-Suarez A, Barriga FM, Jung P, Iglesias M, Cespedes MV, Rossell D, et al. The intestinal stem cell signature identifies colorectal cancer stem cells and predicts disease relapse. Cell Stem Cell. 2011;8(5):511–24. Epub 20110317. doi: 10.1016/j.stem.2011.02.020. PubMed PMID: 21419747.

7. Munoz J, Stange DE, Schepers AG, van de Wetering M, Koo BK, Itzkovitz S, et al. The Lgr5 intestinal stem cell signature: robust expression of proposed quiescent '+4' cell markers. EMBO J. 2012;31(14):3079–91. Epub 20120612. doi: 10.1038/emboj.2012.166. PubMed PMID: 22692129; PubMed Central PMCID: PMCPMC3400017.

8. Neubauer E, Wirtz RM, Kaemmerer D, Athelogou M, Schmidt L, Sanger J, et al. Comparative evaluation of three proliferation markers, Ki-67, TOP2A, and RacGAP1, in bronchopulmonary neuroendocrine neoplasms: Issues and prospects. Oncotarget. 2016;7(27):41959–73. doi: 10.18632/oncotarget.9747. PubMed PMID: 27259241; PubMed Central PMCID: PMCPMC5173108.

9. Kubben FJ, Peeters-Haesevoets A, Engels LG, Baeten CG, Schutte B, Arends JW, et al. Proliferating cell nuclear antigen (PCNA): a new marker to study human colonic cell proliferation. Gut. 1994;35(4):530–5. doi: 10.1136/gut.35.4.530. PubMed PMID: 7909785; PubMed Central PMCID: PMCPMC1374804.

10. Ma Z, Lou S, Jiang Z. PHLDA2 regulates EMT and autophagy in colorectal cancer via the PI3K/AKT signaling pathway. Aging (Albany NY). 2020;12(9):7985–8000. Epub 20200508. doi: 10.18632/aging.103117. PubMed PMID: 32385195; PubMed Central PMCID: PMCPMC7244065.

11. Sasaki N, Sachs N, Wiebrands K, Ellenbroek SI, Fumagalli A, Lyubimova A, et al. Reg4+ deep crypt secretory cells function as epithelial niche for Lgr5+ stem cells in colon. Proc Natl Acad Sci U S A. 2016;113(37):E5399–407. Epub 20160829. doi: 10.1073/pnas.1607327113. PubMed PMID: 27573849; PubMed Central PMCID: PMCPMC5027439.

12. Capdevila C, Miller J, Cheng L, Kornberg A, George JJ, Lee H, et al. Time-resolved fate mapping identifies the intestinal upper crypt zone as an origin of Lgr5+ crypt base columnar cells. Cell. 2024;187(12):3039–55 e14. doi: 10.1016/j.cell.2024.05.001. PubMed PMID: 38848677; PubMed Central PMCID: PMCPMC11770878.

13. Jones JC, Dempsey PJ. Enterocyte progenitors can dedifferentiate to replace lost Lgr5(+) intestinal stem cells revealing that many different progenitor populations can regain stemness. Stem Cell Investig. 2016;3:61. Epub 20161021. doi: 10.21037/sci.2016.09.15. PubMed PMID: 27868043; PubMed Central PMCID: PMCPMC5104576.

14. Rodriguez Sawicki L, Bottasso Arias NM, Scaglia N, Falomir Lockhart LJ, Franchini GR, Storch J, et al. FABP1 knockdown in human enterocytes impairs proliferation and alters lipid metabolism. Biochim Biophys Acta Mol Cell Biol Lipids. 2017;1862(12):1587–94. Epub 20170913. doi: 10.1016/j.bbalip.2017.09.006. PubMed PMID: 28919479; PubMed Central PMCID: PMCPMC5663247.

15. Birchenough GM, Johansson ME, Gustafsson JK, Bergstrom JH, Hansson GC. New developments in goblet cell mucus secretion and function. Mucosal Immunol. 2015;8(4):712–9. Epub 20150415. doi: 10.1038/mi.2015.32. PubMed PMID: 25872481; PubMed Central PMCID: PMCPMC4631840.

16. Gregorieff A, Stange DE, Kujala P, Begthel H, van den Born M, Korving J, et al. The ets-domain transcription factor Spdef promotes maturation of goblet and paneth cells in the intestinal epithelium. Gastroenterology. 2009;137(4):1333–45 e1–3. Epub 20090621. doi: 10.1053/j.gastro.2009.06.044. PubMed PMID: 19549527.

17. Gupta BK, Maher DM, Ebeling MC, Stephenson PD, Puumala SE, Koch MR, et al. Functions and regulation of MUC13 mucin in colon cancer cells. J Gastroenterol. 2014;49(10):1378–91. Epub 20131007. doi: 10.1007/s00535-013-0885-z. PubMed PMID: 24097071; PubMed Central PMCID: PMCPMC3979492.

18. Williams SJ, Wreschner DH, Tran M, Eyre HJ, Sutherland GR, McGuckin MA. Muc13, a novel human cell surface mucin expressed by epithelial and hemopoietic cells. J Biol Chem. 2001;276(21):18327–36. Epub 20010226. doi: 10.1074/jbc.M008850200. PubMed PMID: 11278439.

19. Chan CW, Wong NA, Liu Y, Bicknell D, Turley H, Hollins L, et al. Gastrointestinal differentiation marker Cytokeratin 20 is regulated by homeobox gene CDX1. Proc Natl Acad Sci U S A. 2009;106(6):1936–41. Epub 20090202. doi: 10.1073/pnas.0812904106. PubMed PMID: 19188603; PubMed Central PMCID: PMCPMC2644142.

20. Masumoto Y, Matsuo S, Kinjou N, Narieda Y, Wada M, Fujimoto K. The expression of trefoil factor family member 2 in increased at an acidic pH. Oncol Lett. 2024;27(5):212. Epub 20240315. doi: 10.3892/ol.2024.14345. PubMed PMID: 38572063; PubMed Central PMCID: PMCPMC10988190.
