# Supplementary material for: Genomic profiling of active vitamin D colonic responses in African- and European-Americans identifies an ancestry-related regulatory variant of POLB
Source: PLoS Genet. 2026 Jan 8;22(1):e1011983. doi: 10.1371/journal.pgen.1011983 (PMC12810902; doi:10.1371/journal.pgen.1011983)
Supplement: Attachment — Submitted filename: plosgenetics_vitD_review.docx [file pgen.1011983.s019.docx]

This manuscript represents a substantial and impressive body of work, elegantly integrating both wet-lab experimentation and bioinformatic analyses in a seamless and robust manner.

The study addresses two core issues: characterising transcriptional and genomic responses to 1,25D, and second, assessing potential differences in these responses across two ethnic groups. While the first point is addressed comprehensively, I feel that the second aim (despite being a big focus of the introduction and abstract) receives less emphasis in the main body of the manuscript – with some of the very interesting findings relegated to supplementary figures.

I have split my comments into major and minor criticisms below.

Main criticisms:

1) Referring to your bioinformatic analysis methods, I am not comfortable with the approach of using angsd and ngsRelate to determine relatedness and ancestry admixture from RNAseq and ATACseq data.
   a. I understand that this was done as a QC step, but in my understanding neither of these methods is well-suited for RNAseq due to uneven coverage and allele specific expression/accessibility.
   b. You list several criteria for sample exclusion, but the only one that really matters is whether an individual’s VD and EtOH treatment samples are genetically unrelated (which from supplementary table 1 appears to be only two samples that may have been swapped?). Checking whether expression of CYP24A1 was discordant with treatment labels is a nice positive control and should be presented as such – from my understanding none of the samples were excluded because of this right?
   c. If you want to screen for relatedness, do so using your genotype data – can easily be done with plink2’s implementation of KING-ROBUST. However based on the PCA plots you present it doesn’t seem like any of your participants are related anyway, so I wouldn’t worry about this.
2) In your Methods Model 1, you include *FABP1* expression as a covariate to control for cell type composition, explaining that it was the most highly expressed cell type marker. While *FABP1* is an established enterocyte marker, it seems unusual to rely on a single gene as a proxy for cell type composition. In your supplementary figures, you list several marker genes – why not take a principal component of expression levels of this list?
   a. Speaking of the list of genes shown in Supplementary Figure 1A, you mention that expression was correlated for these genes between treatment and controls but you decided to only plot expression in the controls. I would like to either see a plot or test of this correlation or for you to just include all samples in the figure.
   b. I would also ideally like to see some validation demonstrating that *FABP1* itself is not affected by 1,25D treatment. .
3) The eQTL analyses are actually the main thing I’m concerned about in this paper, all my other criticisms are relatively easy fixes and not that crucial in comparison. It is incredibly difficult to do a meaningful eQTL analysis using such a small sample size.
   a. Testing over 100 million SNP-gene pairs with only 60 samples is definitely underpowered. As mentioned, BRIdGE selects only the top SNPs for each gene, but with the current study design the estimates for top SNPs could be very unstable (many of these top SNPs could reflect noise).
   b. I have similar concerns about the MatrixEQTL analysis, although you were testing much smaller numbers of SNP-gene pairs in separate per-condition analyses, the low sample size causes this to be susceptible to false negatives.
   c. You mention the results between MatrixEQTL and BRIdGE were highly concordant, but only show this visually with a 3 examples. I think a better way would be to show sign concordance (number of pairs with same direction of effect divided by number of all overlapping pairs).
   d. You mention that 49% of eQTLs identified by BRIdGE overlapped with eQTLs reported in GTEX across 48 tissues. First of all, 49% concordance is not great, secondly you should not use the 48 tissue dataset – you should do your comparisons to eQTLs derived from colon tissue, as that is what you purport your organoids to represent.
   e. I would actually recommend removing this analysis entirely. Although it fits with the narrative flow of the paper, providing motivation for subsequent study of *POLB*, the weakness of the study design detracts from an otherwise robust paper.
4) I am not satisfied with the approach in lines 345-353 of using rs2272733 genotype as synonymous with genotype for the indel, even if they are in LD. You should either state this as correlating *POLB* response to the rs2272733 genotype (which is a totally valid thing to do), or actually genotype the deletion itself with a quick PCR.
5) It is mentioned in passing that there were 77 genes that differ between ancestries in their response to 1,25D – I think this result should be better highlighted as it is a significant finding in the paper. Perhaps in your discussion?

Minor criticisms:

1) I’m not understanding the discrepancy between number of participants who were biopsied (N=60) and participants who were genotyped (N=64)? Unless line 521 is a typo and you meant 3 participants did not have available biopsies – then there is still a discrepancy of 60 vs 61 – please double check this.
2) Figure 1B would look clearer if you grey out the dots that are less than an absolute LFC > 1.5 (can be a darker grey than the points that are insignificant).
3) When identifying enrichment of the vitamin D response element in DA peaks, you use a degenerate consensus motif allowing variability at multiple positions – allowing for analysis of a family of VDR-RXR binding sites rather than a single fixed sequence. I would like for this to be more explicitly clarified in the main text.
4) Do you have any explanation for the very clear segregation between samples along PC1 of Supplementary Figure 1C?
5) This may be nitpicky, but for the sake of reproducibility I would feel more comfortable seeing the size markers in the gel in Figure 5D.
6) It would be easier to follow if you added titles to your graphs for Figures 5e-g.
7) Typo in supplementary table 1 – tissue column should be sex.
8) I take issue with the statement in line 268-269 that only individuals with the ancestral haplotype showed 1,25D expression and accessibility responses. You are using rs2272733 as a proxy for the entire haplotype (which is all you can feasibly do, given the sample size) – but this is not the same as testing the haplotype against *POLB* response to treatment.
