# Supplementary material for: Genomic profiling of active vitamin D colonic responses in African- and European-Americans identifies an ancestry-related regulatory variant of POLB
Source: PLoS Genet. 2026 Jan 8;22(1):e1011983. doi: 10.1371/journal.pgen.1011983 (PMC12810902; doi:10.1371/journal.pgen.1011983)
Supplement: Attachment — Submitted filename: Responses to reviewers PLOS Genetics FINAL.docx [file pgen.1011983.s021.docx]

**Response to Reviewers**

**Reviewer #1:** This is an interesting and thorough study of the effect of 1,25D on colon organoids, with emphasis on the POLB findings. In particular, this reviewer appreciated the multi-omic approach and thoughtful consideration of ancestry. Below are concerns about the overall approach, as well as some comments related to interpretation.

**Response**: We thank the reviewer for these positive comments. We are grateful for the reviewer’s constructive criticisms and believe our manuscript has been strengthened by their comments and suggestions.

1) I am a bit confused by the information presented in Figures 1D and 1F the 1D, the authors state ~4000 peaks were significantly associated with 1,25D treatment. But in 1F, the number by ancestry (one alone or both) is only ~500, as the legend states.

**Response:** Thank you for this comment. For the volcano plot depicted in Figure 1D, we included ATAC data for all samples that met quality control filters (n=25 lines). For the ancestry analysis, we evaluated each population separately (n=12 AA and 13 EA) and reported on DA peaks in each population or both. Due to the smaller sample sizes in the per-ancestry analysis, we had decreased power to detect DA peaks which explains these differences. To address this comment, we have included sample sizes in the Figure 1 legend (p37) and text for clarity (p7, line 148 and p10, line 219).

2) For eQTL analysis, the author states that they used BRIdGE and MatrixQTL to identify eQTLs; however, there is no mention of multiple testing correction commonly done in eQTL analysis. An FDR (Or posterior probability cut-off) is not sufficient for the number of SNPs tested, of the number of genes assessed. More up-to-date methods have now been created to assess Context-specific eQTLs (Meta-Tissue, MashR).

**Response:** Based on the reviewer’s recommendation, we redid eQTL analyses using the multivariate adaptive shrinkage R (mashr) package to find context-specific response-eQTLs (reQTLs) using thresholds defined by Urbut S et al (PMID 30478440). We have updated the Methods and Results sections to reflect these new analyses and results. We note that these new analyses did not change results regarding our main finding of vitamin D responses of *POLB.*

**METHODS** (p33, line 801)*:* “To find condition-specific response-eQTLs, the program mashr [25] (v0.2.79) was run. Approximately 200,000 randomly chosen eQTLs pruned for LD were used to establish the null correlation matrix and the canonical set of covariance matrices alone were used for fitting the mashr model. Following the recommendation in Urbut et al, an eQTL was considered a response-eQTL if 1) its local false sign rate (lfsr)<0.05 in either treatment condition and 2) either the effect size posterior means had the same sign in the two conditions and differed by a factor > 2 or had opposite signs.”

**RESULTS** (p10, line 224): “To identify possible genetic contributions to the variation in 1,25D transcriptional responses, we mapped *cis-*expression quantitative trait loci (eQTLs) separately within each treatment condition using the program fastQTL [24]. We then used the Multivariate Adaptive Shrinkage in R package mashr [25] to identify genotype by DE interaction response-eQTLs (reQTLs). At a local false sign rate (lfsr) <0.05 in at least one treatment condition, we identified 41 genes with an reQTL with a >2x larger posterior mean effect size (pm) in 1,25D, 21 genes with an reQTL with a >2x larger pm in control, and 230 genes with an reQTL that had significantly nonzero effect sizes in at least one condition but with opposite signs (**Supplementary Table 7; Supplementary Figure 5a & b**).”

3) In the eQTL analysis, no covariate (age, sex, etc) seems to have been added to the analysis. Were both ancestors mapped together? This may pose a real issue if no PC were added. No SVA or PEER was included to correct for hidden variables in gene expression.

**Response:** To address this concern, we included more information about covariates per the reviewer’s suggestions. Ancestries were mapped together and the first 3 genetics PCs were regressed out for *cis-*eQTL mapping. In addition, surrogate variable analysis identified one significant surrogate variable in the residual expression values which was added as a covariate to account for hidden variables. These analyses were included in the revised methods section as detailed below:

**METHODS**:

p28, line 667: “**Genetic relatedness and ancestry estimates.** Genetic ancestry proportions were estimated with the program ADMIXTURE [53] (v1.3.0) using approximately 255,000 imputed SNPs that were pruned for linkage disequilibrium and filtered for a minor allele frequency greater than 0.05 using PLINK2 [54] (v2.00) commands –indep-pairwise 50kb 1~~0~~ 0.2 and –maf 0.05. The genotype data were then merged with the genotype data from 10 CEU, 10 YRI, and 10 CHB from the 1000 Genome Project. ADMIXTURE was run with k=3 to capture African, European, and possibly Native American or Asian ancestry components of individuals who self-identified as either non-Hispanic White or Black/African American. Principal component analysis (PCA) was performed on the same set of SNPs using PLINK2 with the --pca command. Genetic relatedness was estimated with the same set of SNPs and the PLINK2 command –make-king-table. No pair of individuals was found to have a KING kinship coefficient greater than 0.019.”

p33, line 791: ***“cis-eQTL analyses.*** *cis*-eQTL mapping was performed separately on samples from each treatment condition using the program fastQTL [24](v2.184_gtex). The raw expression data of protein coding genes was first log-transformed and normalized for library size using the vst function of DESeq2. The data was then quantile normalized across all samples from both treatment conditions and, to control for genetic ancestry, the first 3 genetic PCs (see **Genetic relatedness and ancestry estimates**.) were regressed out. The sva function from the sva R package [64] (v3.52.0) found a single significant surrogate variable in the residual expression values, and this was added as a covariate in the fastQTL model.”

4) The authors focus on POLB because of the difference in transcriptional response with treatment. In the LFC on ATAC seq peak near this locus, only 9 to 10 points are shown. Why are there so few samples in this analysis? The methods are stated 12-13 in each group.

**Response:** We thank the reviewer for catching this omission. We have updated Figure 3B to reflect the correct number of samples.

5) 3 models of DE analysis are described in the methods, but only one is shown in the results (Model 1). The other. Models are very interesting, and the results of those should also be presented.

**Response:** Per the reviewer’s suggestion, we have included results from Model 2A and 2B in the revised manuscript. We have explicitly called out the models in the results section to highlight these findings. A new Supplementary Figure 4 was added with the expanded results from the two models, and Table 2 was updated with new numbers.

**RESULTS** (p9, line 201): “To identify individual genes with ancestry-related differences in 1,25D treatment responses, we applied a mixed model similar to that used to identify DE genes but with an added treatment by ancestry interaction term. This model yielded highly concordant results regardless of whether ancestry was approximated by a dichotomous population variable or a continuous variable of African ancestry proportion (see Methods *Models 2a & b*), as demonstrated by a strong correlation between interaction term z-scores (**Supplementary Figure 4**). We used a nested approach that focused on the subset of genes that had shown both significant differential expression by 1,25D treatment and by ancestry (n=81 genes). A total of 8/81 (9.9%) genes showed significant differences in treatment response between AA and EA (**Table 2**), with four genes (*AMACR*, *TMEM179B*, *SRMS* and *AKAP5*) DE only in AA, one gene (*HS6ST1*) DE only in EA, and three genes (*EPB41L1*, *KYNU*, and *POLB*) DE in both populations, all having 1,25D AA/EA responses in the same direction but with significantly different effect sizes.”

6) It would be interesting to know if the eQTL identified after treatment are located in DA genomics regions, with the idea that some SNPs may not exert a regulatory effect until they are located within open chromatin.

**Response:** Thank you for this helpful suggestion. To address this, we assessed overlap of response-eQTLs (reQTLs) from the mashR analysis with DA regions. Six DA regions were identified associated with 4 genes including *POLB.* To further evaluate whether the DA regions mediate the relationship between reQTL and differential gene expression, we applied a Bayesian mediation analysis model, bmediatrR, and found that the *POLB* indel exhibited a high posterior probability supporting partial or complete mediation by the DA region. These new results have been included in the revised manuscript.

**RESULTS** (p11, line 247): “The significant reQTLs were intersected with the DA regions to determine whether any might exert a direct causal effect on eGene regulation through changes in chromatin accessibility. Six DA regions were found to harbor 7 reQTLs associated with 4 eGenes: *POLB* (8:42190387:A:<CN0>:42194352), *FER1L6* (rs4242349), *SGMS2* (rs28411912; rs28626074; rs28585029), and *ARHGEF28* (rs2112161; rs4703608). All four of these eGenes were found to be significantly upregulated by 1,25D. The reQTL associated with *POLB* is an approximately 4 kb indel upstream of the *POLB* TSS that encompasses the entire DA peak. To evaluate whether the DA regions mediate the relationship between reQTL genotype and eGene differential expression, we used bmediatrR, a Bayesian model selection framework for mediation analysis. Of the 7 reQTLs tested, the *POLB* indel exhibited appreciable posterior probability (76%) supporting either complete or partial mediation by the DA region (**Supplementary Figure 6a-g**).

7) Missing from the discussion is a clear delineation of limitations, such as the use of organoid cultures, which may differ from colonic tissue in vivo, the relatively small sample size, and the power to detect association inherent in the study, especially once divided by ancestry.

**Response:** We thank the reviewer for this suggestion. The revised discussion includes a new paragraph describing strengths and limitations of the study.

**DISCUSSION** (p22, line 511): “Strengths of this study include a paired treatment design that controls for confounders, focus on 1,25D responses on the human colonic epithelium, multi-level treatment response data on chromatin accessibility and differential gene expression in organoid lines from the same individuals, and inclusion of individuals of different ancestral backgrounds. We acknowledge several limitations including assessment of epithelial responses *in vitro* that might not completely recapitulate responses *in vivo*, lack of data on responses in stromal or immune cells that could have relevance for vitamin D’s biological actions in the human colon, and a moderate sample size that could lack sufficient power to detect all treatment responses especially when divided by ancestry. Despite these limitations, the study nonetheless identified inter-individual 1,25D responses including the novel ancestry-related regulatory region for *POLB* responses.”

8) People of African Ancestry are known to have lower levels of Vitamin D as well as higher levels of VDR. How would these differences relate to your findings? While genetic regulation is interesting, would the long-noted difference long noted blunt the findings?

**Response:** We thank the reviewer for these important questions. While lower serum vitamin D levels in individuals of African ancestry are well-described, our study asked the question whether genomic responses to the *same* amount of active vitamin D (1,25D) differ between individuals. In the present study, we did not have serum vitamin D levels for the individuals from whom organoids were derived. However, in our previous work (PMID 28163244), we showed that controlling for vitamin D serum levels as covariates in the assessment of differential expression in response to 1,25D did not alter transcriptional responses appreciably; thus, we do not think that serum vitamin D plays a significant role in 1,25D colonic responses *in vitro.* Future studies could assess colonic responses to varying doses of inactive vitamin D to directly answer the reviewer’s question.

Regarding the question about VDR differences by ancestry, we found that, while 1,25D treatment showed a small but significant downregulation of the gene encoding *VDR*, no differences in responses were found between populations. These results have been included in the revised manuscript.

**RESULTS:**

p7, line 129: “The gene encoding VDR showed a small but significant downregulation with 1,25D treatment (LFC=-0.35; FDR=6.2x10^-17^).

p10, line 212: “The gene encoding VDR showed no significant difference in differential expression between populations (LFC_AA_-LFC_EA_=-0.038; FDR=0.832).”

Minor Comments
1) As a result, the authors state that they controlled for Ancestry (line 102) in the DE analysis (Model 1). I believe they mean population as Ancestry is controlled for in Model 3. (did they mean 2b)

**Response:** This was clarified in the revised manuscript.

Reviewer #2

This manuscript represents a substantial and impressive body of work, elegantly integrating both wet-lab experimentation and bioinformatic analyses in a seamless and robust manner.

The study addresses two core issues: characterising transcriptional and genomic responses to 1,25D, and second, assessing potential differences in these responses across two ethnic groups. While the first point is addressed comprehensively, I feel that the second aim (despite being a big focus of the introduction and abstract) receives less emphasis in the main body of the manuscript – with some of the very interesting findings relegated to supplementary figures.

**Response:** We thank the reviewer for these important comments that have motivated us to perform additional analyses, especially for the eQTL mapping, that we believe have substantially strengthened the paper.

I have split my comments into major and minor criticisms below.

Main criticisms:

1) Referring to your bioinformatic analysis methods, I am not comfortable with the approach of using angsd and ngsRelate to determine relatedness and ancestry admixture from RNAseq and ATACseq data.
   a. I understand that this was done as a QC step, but in my understanding neither of these methods is well-suited for RNAseq due to uneven coverage and allele specific expression/accessibility.

**Response:** We thank the reviewer for this concern. As correctly stated by the reviewer, this analysis was performed as a conservative quality control step to determine potential swaps in the RNA-seq data. The intention was not to determine overall relatedness or ancestry from these data.

To address the reviewer’s concern, we performed overall relatedness analyses, in addition to ancestry estimation, on the genotype data. This has been added to the methods section.

**METHODS** (p28, line 675): “Genetic relatedness was estimated with the same set of SNPs and the PLINK2 command –make-king-table. No pair of individuals was found to have a KING kinship coefficient greater than 0.019.”

b. You list several criteria for sample exclusion, but the only one that really matters is whether an individual’s VD and EtOH treatment samples are genetically unrelated (which from supplementary table 1 appears to be only two samples that may have been swapped?). Checking whether expression of CYP24A1 was discordant with treatment labels is a nice positive control and should be presented as such – from my understanding none of the samples were excluded because of this right?

**Response:** We appreciate the reviewer’s comments. In the revised manuscript, we have more clearly delineated the quality control steps for both RNA-seq and ATAC-seq data in line with the reviewer’s suggestions. The quality control steps have also been included in more detail in **Supplementary Table 1**.

**METHODS** (p29, line 688)**:** “An individual’s paired RNA-seq samples were removed from downstream analyses if any of the following were found to be true: (1) the 1,25D and control treatment samples were genetically unrelated; (2) either treatment sample was genetically related to a sample from a different individual; (3) either treatment sample showed an ancestry proportion dissimilar to that estimated from the genotype data. After applying these filters, 53 lines were available for transcriptome profiling.”...“As an additional quality check, the expression of the canonical vitamin D responsive gene *CYP24A1* was observed to be significantly upregulated in all 53 lines.”

**METHODS** (p31, line 758): “Similar to the RNA-seq samples, ATAC samples were checked for sample relatedness and ancestry proportion using the ngsRelate and fastNGSadmix tools, respectively, and individual’s ATAC-seq samples were removed from further downstream analysis if any of the following applied: (1) the individual’s 1,25D and control treatment samples were genetically unrelated; (2) either treatment sample was genetically related to a sample from a different individual; or (3) either treatment sample showed an ancestry proportion dissimilar to that estimated from the genotype data.”...”As an additional quality check, a significant DA peak with LFC(VD/EtOH)>1 was observed in the *CYP24A1*promoter region of all 25 lines.”

c. If you want to screen for relatedness, do so using your genotype data – can easily be done with plink2’s implementation of KING-ROBUST. However based on the PCA plots you present it doesn’t seem like any of your participants are related anyway, so I wouldn’t worry about this.

**Response:** As detailed in the response in 1a, we have followed the reviewer’s suggestion and performed the relatedness analysis using the genotype data. As suggested by the reviewer, we did not find evidence of relatedness between participants.

2) In your Methods Model 1, you include *FABP1* expression as a covariate to control for cell type composition, explaining that it was the most highly expressed cell type marker. While *FABP1* is an established enterocyte marker, it seems unusual to rely on a single gene as a proxy for cell type composition. In your supplementary figures, you list several marker genes – why not take a principal component of expression levels of this list?

**Response:** We appreciate the reviewer’s comments regarding cell composition. To more comprehensively control for cell composition, we leveraged a single cell RNA-sequencing dataset and used CIBERSORTx to create a signature matrix to impute relative cell type abundance from the expression data. Given that enterocytes (both early and mature cells) are cell types of interest and make up the majority of cells in organoid cultures, we included the total fraction of early enterocytes and enterocytes as a covariate in the models.

**METHODS** (p29, line 705): “Single cell analysis of a single organoid line after 24, 48 and 72 hours in differentiation and growth media was performed previously (**Supplementary Methods**). The cell populations from these pooled libraries included stem cells, proliferating stem cells, early enterocytes, enterocytes and goblet cells (**Supplementary Figure 1a & b**). The web-based application CIBERSORTx [65] was used to create a signature matrix from the single cell expression data and impute the relative cell type abundance from the bulk expression data. The total fraction of early enterocytes and enterocytes was included as an additional covariate in the models to control for potential confounding by cell type composition among organoids. Of note, no differences in cell type composition were observed either by treatment or population (**Supplementary Figure 1c**).

a. Speaking of the list of genes shown in Supplementary Figure 1A, you mention that expression was correlated for these genes between treatment and controls but you decided to only plot expression in the controls. I would like to either see a plot or test of this correlation or for you to just include all samples in the figure.
b. I would also ideally like to see some validation demonstrating that *FABP1* itself is not affected by 1,25D treatment.

**Response:** To address these specific concerns, we assessed for differences by treatment and ancestry for the early enterocyte and enterocyte fractions. No differences in cell type composition were noted by treatment or population. This has been included in **Supplementary Figure 1c**.

3) The eQTL analyses are actually the main thing I’m concerned about in this paper, all my other criticisms are relatively easy fixes and not that crucial in comparison. It is incredibly difficult to do a meaningful eQTL analysis using such a small sample size.

**Response:** We thank the reviewer for this main concern. The eQTL mapping approach was also raised as a concern by Reviewer 1 (see comment #2).

To address these concerns, we have redone mapping to identify context-specific response eQTLs (reQTLs) using the multivariate adaptive shrinkage (mashR) method developed by Urbut et al (PMID 30478440). As detailed in the Urbut et al paper, compared to other approaches, mashR increased power as demonstrated in an example using a moderately-sized cohort (n=44), notably a sample smaller than that used in our study.

Using thresholds recommended in the Urbut et al paper (local false sign rate, lfsr, <0.05 in at least one condition), we identified that ~2% (264/13,919) of the genes tested had 2-fold or greater posterior mean (pm) effect size in at least one treatment condition. Of these, 41 reQTLs had higher pm in 1,25D, and 21 reQTLs had higher pm in control. A total of 230 reQTLs had significantly nonzero effect sizes in at least one condition but with opposite signs.

The revised reQTL approaches and analyses have been included in the Methods and Results. See responses to Reviewer 1, comment #2 for changes in the text. We have also removed Figure 2 and now include context-specific reQTL results as Supplementary materials.

a. Testing over 100 million SNP-gene pairs with only 60 samples is definitely underpowered. As mentioned, BRIdGE selects only the top SNPs for each gene, but with the current study design the estimates for top SNPs could be very unstable (many of these top SNPs could reflect noise).

**Response:** In addition to redoing the eQTL analyses using mashr which increases power, we also changed parameter thresholds to address issues of multiple testing and power. Specifically, we changed the minor allele frequency (MAF) threshold to >10% and changed the genomic windows to 100KB from 500KB for fastQTL. These filters resulted in approximately 5.6 million gene-variant pairs for mapping. These modifications have been included in the revised methods.

**METHODS** (p33, line 798): “Autosomal bi-allellic variants tested were those having a MAF>0.10 and falling within a 100 kb window of each tested gene’s transcription start site. After applying these filters, there were approximately 5.6 million gene-variant pairs for analysis.”

b. I have similar concerns about the MatrixEQTL analysis, although you were testing much smaller numbers of SNP-gene pairs in separate per-condition analyses, the low sample size causes this to be susceptible to false negatives.

**Response:** We acknowledge a small sample size but believe that we have identified true positive results using our methodologies. It is possible that additional reQTLs have been missed and could be identified with a larger sample size. We have included this limitation in a new paragraph in the discussion. See Reviewer 1, comment #7 for this new text.

c. You mention the results between MatrixEQTL and BRIdGE were highly concordant but only show this visually with 3 examples. I think a better way would be to show sign concordance (number of pairs with same direction of effect divided by number of all overlapping pairs).

**Response:** Given that eQTL mapping was performed using mashr, this analysis has been deleted from the revised manuscript.

d. You mention that 49% of eQTLs identified by BRIdGE overlapped with eQTLs reported in GTEX across 48 tissues. First of all, 49% concordance is not great, secondly you should not use the 48 tissue dataset – you should do your comparisons to eQTLs derived from colon tissue, as that is what you purport your organoids to represent.

**Response:** We thank the reviewer for this comment. To address this, we performed additional analyses in which significant reQTL mashr results (lfsr<0.05) were intersected with significant GTEx eQTLs [metasoft posterior probability (m-value) >0.90] from sigmoid and/or transverse colon tissue. We found an overlap of 78.6% between significant GTEx eQTLs in either colon tissue and significant reQTLs in both treatment conditions. When restricting the overlap to only significant reQTLs in control treatment, the overlap with GTEx eQTLs increased to 88.5%. Interestingly, the overlap between significant reQTLs in 1,25D showed an overlap of only 37.7% highlighting the value of performing context-specific reQTL mapping to identify regulatory variants of treatment responses *not* captured by GTEx. These findings are included in the revised manuscript.

**METHODS** (p33, line 807)**:** “The significant results from mashr were overlapped with the roughly 3.5 million eQTLs from sigmoid and transverse colon tissue listed in the 48 cross-tissue metasoft analysis of GTEx with metasoft posterior probability (m-value) >0.90 in either tissue. We found that 53.0% of listed GTEx eQTLs have m>0.90 in either sigmoid or transverse colon tissue; when conditioning on these eQTLs with m>0.90 in at least one of the two tissues, 60.9% have m>0.90 in both tissues. For our tested eQTLs overlapping with those in the GTEx metasoft database, m>0.90 in either colon tissue for 78.6% of 31,533 eQTLs with lfsr<0.05 in both vehicle control and 1,25D; for 88.5% of the 295 eQTLs with lfsr<0.05 only in vehicle control; and for 37.7% of the 875 eQTLs with lfsr<0.05 only in 1,25D.”

e. I would actually recommend removing this analysis entirely. Although it fits with the narrative flow of the paper, providing motivation for subsequent study of *POLB*, the weakness of the study design detracts from an otherwise robust paper.

**Response:** To address the reviewer’s concern, we have removed Figure 2 from the revised paper. We have updated the methods and results to reflect the revised mapping strategy that we believe has strengthened the paper. As pointed out by the reviewer, it was these analyses that first identified the indel regulatory variant near *POLB;* thus, we believe it is important to include this approach in the paper for context.

4) I am not satisfied with the approach in lines 345-353 of using rs2272733 genotype as synonymous with genotype for the indel, even if they are in LD. You should either state this as correlating *POLB* response to the rs2272733 genotype (which is a totally valid thing to do), or actually genotype the deletion itself with a quick PCR.

**Response:** We thank the reviewer for this comment. As presented in the revised manuscript, we found that the indel genotype was imputed with a posterior probability of >0.942 in all samples. Specifically, for the organoid lines used for validation studies, the imputation posterior probability was >0.999 in every line. Given perfect LD between the genotyped SNP rs2272733 and the imputed indel (r^2^=1), we believe our inference is valid. We further validated indel insertion carrier status of our samples using ATAC-seq coverage of the region.

In response to the reviewer’s comment, we have added more details to the methods and results sections as well as highlighting this in the relevant Figure legends.

**METHODS** (p35, line 844)**: “*Inference of POLB indel genotype*:** The genotype of the indel (nssv16196380) proximal to the POLB promoter is imputed in our samples using the 1000 Genome Project Phase 3 dataset as a reference. Overall, the posterior probability for indel genotype imputation was >0.942. For the lines used for validation, the posterior probability for the indel was >0.999. We also more directly infer indel genotype from the genotyped SNP rs2272733 because the two are in perfect LD (r^2^=1) in the 1KG CEU and YRI samples, as well as in our samples. We also validated genotype status of samples inferred to be homozygous deletion from ATAC-seq coverage of the region.”

**RESULTS** (p16, line 385): “***Genotype-specific POLB*** ***vitamin D responses and VDR binding in indel region.*** To confirm that the *POLB* responses to 1,25D correlated with indel genotype, we identified organoid lines with homozygous deletion (D/D), heterozygotes (I/D) and homozygous insertion (I/I) based on high-quality imputation results and inferred by rs2272733 genotype which is in perfect LD (r^2^=1) with the indel.”

**FIGURE 4 & 5 LEGENDS**: “For these analyses, the indel genotype was defined by high-quality imputed genotype data (posterior probability for imputed genotype >0.999). Imputed genotypes matched genotype of rs2272733, a SNP in perfect linkage disequilibrium (r^2^=1) with the indel in 1KG CEU and YRI.”

5) It is mentioned in passing that there were 77 genes that differ between ancestries in their response to 1,25D – I think this result should be better highlighted as it is a significant finding in the paper. Perhaps in your discussion?

**Response:** We thank the reviewer for this comment. This has been added to the discussion (p20).

Minor criticisms:

1) I’m not understanding the discrepancy between number of participants who were biopsied (N=60) and participants who were genotyped (N=64)? Unless line 521 is a typo and you meant 3 participants did not have available biopsies – then there is still a discrepancy of 60 vs 61 – please double check this.

**Response:** Thank you for noting this discrepancy. The genotyping was performed using DNA from 64 samples. However, this included participants whose organoids were not part of the current study. We have clarified that all 60 participants included in this study were genotyped (p24).

2) Figure 1B would look clearer if you grey out the dots that are less than an absolute LFC > 1.5 (can be a darker grey than the points that are insignificant).

**Response:** This has been done per the reviewer’s suggestions.

3) When identifying enrichment of the vitamin D response element in DA peaks, you use a degenerate consensus motif allowing variability at multiple positions – allowing for analysis of a family of VDR-RXR binding sites rather than a single fixed sequence. I would like for this to be more explicitly clarified in the main text.

**Response:** Thank you for this observation. We have clarified this in the results.

**RESULTS** (p8, line 172): “The motif for the vitamin D response element (VDRE), defined by the degenerate consensus motif of VDR and its partner RXR, was present in 19.7% of DA peaks compared to only 2.3% of background peaks (p=1.0x10^–481^; **Supplementary Table 6**). It should be noted that this analysis used the degenerate consensus motif as defined in *Homer* allowing for variability at multiple positions and a family of VDR-RXR binding sites rather than a single fixed sequence.”

4) Do you have any explanation for the very clear segregation between samples along PC1 of Supplementary Figure 1C?

**Response:** PC1 separation is because we included sex chromosomes in this analysis. This has been added to the supplementary figure legend.

5) This may be nitpicky, but for the sake of reproducibility I would feel more comfortable seeing the size markers in the gel in Figure 5D.

**Response:** The ladder has been added to the new Figure 4d per the reviewer’s suggestion.

6) It would be easier to follow if you added titles to your graphs for Figures 5e-g.

**Response:** Given limited space in the figure itself for descriptive titles, we separated out the individual panels in the figure legend for clarity. We hope this addresses the reviewer’s concern adequately.

7) Typo in supplementary table 1 – tissue column should be sex.

**Response:** This has been corrected.

8) I take issue with the statement in line 268-269 that only individuals with the ancestral haplotype showed 1,25D expression and accessibility responses. You are using rs2272733 as a proxy for the entire haplotype (which is all you can feasibly do, given the sample size) – but this is not the same as testing the haplotype against *POLB* response to treatment.

**Response:** This comment has been addressed in the results and included in the relevant figure legends for clarity.

**RESULTS** (p13, line 307): “Taken together, 1,25D responses of *POLB* were of interest due to: 1) ancestry-related transcriptional response differences, 2) a long-range haplotype tagged by a significant response *cis*-eQTL, rs2272733, with large frequency differences between populations, 3) a proximal 4 kb indel harboring a DA region that likely mediates between genotype and *POLB* differential expression, 4) significant non-zero 1,25D expression and accessibility responses only in individuals with the ancestral tagging allele of rs2272733, 5) the gene’s role as a key polymerase in base excision repair [31] implicated in tumorigenesis [32-34], and 6) association of rs2272733 with colorectal cancer [30]. These findings suggest that an ancestral alteration in *POLB* regulation might be an important driver of inter-ethnic differences in responsiveness to 1,25D, with potentially significant consequences for disease risk.”
